# Hypokalemic Periodic Paralysis: An Atypical Presentation of Non-autoimmune Hypothyroidism With Distal Renal Tubular Acidosis

**DOI:** 10.7759/cureus.24046

**Published:** 2022-04-11

**Authors:** Humaira Achakzai, Sadia Khan, Qazi Kamran Amin, Naseer Ahmed, Safa Anwar

**Affiliations:** 1 Internal Medicine, Rehman Medical Institute, Peshawar, PAK; 2 General Medicine, Rehman Medical Institute, Peshawar, PAK; 3 Cardiovascular Sciences, University of Verona, Verona, ITA; 4 Department of Biological and Biomedical Sciences, Aga Khan University Hospital, Karachi, PAK

**Keywords:** drta, electrolyte imbalance, limb weakness, quadriplegia, hypo kpp, case report, distal renal tubular acidosis, non autoimmune hypothyroidism, periodic paralysis, hypokalemia

## Abstract

Hypokalemic periodic paralysis (hypo KPP) is a rare form of autosomal dominant channelopathy characterized by muscular weakness and paralysis caused by decreased potassium levels. Precipitating factors are a diet rich in starches and sweets, and rest after an unusual degree of exercise. Paralytic attacks are more common between the ages of 15 and 40 years. The presentation can be a total paralysis or severe quadriplegia or mild weakness in certain group of muscles. During the acute episode of weakness proximal muscles are involved initially with gradual spread to the distal muscles. Deep reflexes are decreased or absent but the cognitive functions and sensory systems are intact. The paralysis may last for few hours to several days, but recovery is usually sudden in most patients. Hypo KPP is usually associated with thyroid disorders and distal renal tubular acidosis (DRTA). Here we report a case of young female patient who presented in emergency with two days history of weakness of all four limbs. The patient also had two episodes of similar illness in the last two and half years. On examination she had decreased tone and power in all four limbs with absent deep tendon reflexes, and plantar reflexes were down going bilaterally. On initial laboratory workup, patient was diagnosed to have hypokalemic, hyperchloremic metabolic acidosis with alkaline urine secondary to hypothyroidism. Features of hypokalemia with metabolic acidosis and failure to acidify urine was consistent with DRTA. Intravenous potassium chloride and bicarbonate replacement resulted in biochemical and clinical improvement.

## Introduction

Hypokalemic periodic paralysis is a rare type of periodic paralysis, with an estimated prevalence of 1 in 100,000 [1]. In distal renal tubular acidosis (DRTA), the kidneys fail to acidify urine, either due to secretory defect in pumps or increased luminal membrane permeability defect in renal tubular cells, which is in turn due to failure of hydrogen-potassium ATPase or hydrogen ATPase pump [2]. Distal renal tubular acidosis has been previously described to be closely associated with autoimmune diseases like systemic lupus erythematosus, Sjogren’s syndrome, hyperglobulinemia, and autoimmune hypothyroidism. The cause of DRTA In hypothyroidism is usually of autoimmune origin, but a non-autoimmune cause of DRTA in hypothyroidism is extremely rare [3]. Only two cases of hypokalemic periodic paralysis associated with non-autoimmune hypothyroidism with DRTA have been reported so far [4]. Periodic paralysis associated with hypokalemia is an atypical presentation in non-autoimmune hypothyroidism. This is a case of distal renal tubular acidosis in a patient with non-autoimmune hypothyroidism presenting as hypokalemic periodic paralysis.

## Case presentation

A 30-year-old female patient presented to the emergency department at Rehman Medical Institute, Peshawar, in 2017 with chief complaints of both upper and lower limb weakness. Headache and progressive drowsiness started two days prior to her presentation. The weakness was sudden in onset and progressed to generalized flaccid paralysis. The patient was living with her husband and four children, with a good socioeconomic background. On examination the patient had normal build, but was ill-looking, in respiratory distress, and had slight confusion with Glasgow Coma Scale of 14/15. Her blood pressure was 130/70 mmHg, pulse 98 beats/min, respiratory rate of 25 to 28 per minute, oxygen saturation of 96% at room air and temperature 98 degrees Fahrenheit. There was no pallor, no proptosis, facial puffiness or edema. Head and neck examination demonstrated no palpable thyroid gland and ophthalmoplegia or lid retraction. Both upper and lower limbs examination showed no signs of atrophy, fasciculation, tremor, spasticity, but all four limbs showed hypotonia with grade 4 quadriparesis and absent deep tendon reflexes. The cranial nerves were all intact, plantar reflexes were down going bilaterally (Babinski sign negative). Other systemic examinations like cardiovascular and gastro-Intestinal were unremarkable.

The patient's past medical history was unremarkable for any viral illness, drug intake, vomiting and loss of consciousness. However, she had had two similar episodes of paralysis in the last two and half years that she recovered from in two to three days with simple correction of potassium levels, managed at a local health care facility. There was no history of smoking, alcohol, or drug abuse. There was no past medical history of any autoimmune disease, hypothyroidism, or neuromuscular disorder.

The patient was initially stabilized in the emergency department and was then shifted to an intensive care unit. Initial laboratory workup showed severe hypokalemia of 1.90 mmol/L, hypernatremia 151.6mmol/l, a raised white blood cell count 25 x 10^3/uL, creatinine 1.26mg/dl, chloride 117.7mmol/l with elevated creatine phosphokinase (CPK) 1870u/l and the arterial blood gases showed severe metabolic acidosis with pH 7.21, partial pressure of carbon dioxide (PCO2) 26.5, bicarbonate (HCO3) 10.6mmol/l. Thyroid function test revealed low T4 5.15pmol/l, T3 0.651nmol/l and high thyroid stimulating hormone (TSH) 46.51mIU/l which was concerning for hypothyroidism. Urine routine examination showed pH of 8.0 and ++ proteins. Electrocardiogram showed a normal sinus rhythm, long QT interval without any other abnormality. Chest Xray radiograph, ultrasound abdomen and computed tomography of abdomen were all normal. Electromyography and nerve conduction studies were done to rule out any neurological causes of the paralysis, but the report came out to be normal.

Keeping in view the above findings a provisional diagnosis of hypokalemic periodic paralysis with hypothyroidism was made. She was treated with intravenous potassium chloride infusion (KCL), sodium bicarbonate and oral thyroxin tablet 200mg stat, followed by 150mcg/day. Despite our efforts hypokalemia and metabolic acidosis could not be corrected, furthermore her urine failed to acidify on urine routine examination (Table 1). Hence, the possible cause of hypokalemia was found to be of renal origin. There were no symptoms or laboratory findings of Bartter or Gitelman syndrome. The absence of hypertension, normal serum aldosterone level and normal adrenal gland on CT abdomen excluded hyperaldosteronism. All the primary causes of periodic paralysis due to hypokalemia with metabolic acidosis were ruled out, except secondary causes. In secondary causes the most plausible explanation for her presentation was distal renal tubular acidosis. Furthermore, features of DRTA such as polyuria, polydipsia, acidotic breath and laboratory findings like urine low pH, hyperchloremia (117mmol/l) during the acute paralysis were present. Anti thyroid peroxidase antibodies (anti-TPO) were negative, which ruled out autoimmune hypothyroidism; hence a diagnosis of non-autoimmune hypothyroidism with distal renal tubular acidosis was made.

**Table 1:** Shows the daily trend in all the relevant laboratory investigations. Normal values with units are given in brackets in the first column after the investigation name. PCO2: partial pressure of carbon dioxide, HCO3: bicarbonate

LABS	DAY1	DAY2	DAY3	DAY4	DAY5	DAY6	DAY7	DAY9	DAY10	DAY11	DAY12	FOLLOW UP AFTER 10 DAYS
Hemoglobin (12.5–16.5 g/dl)	15	15.3	14.2	9.9	11.5	-	-	-	-	-	-	-
Total Leukocyte Count (4–11 x 10^3/uL)	25	14.63	24.65	17.22	18.17	-	-	-	-	-	-	-
Platelet (150–450 10^9/L)	340	326	349	850	252	-	-	-	-	-	-	-
Sodium (135–148 mmol/l)	151	162	171	167	159	139	147	-	140	143	141	135
Potassium (3.6–5.2 mmol/l)	1.9	2.13	2.86	3	3.16	3.19	3.51	-	4.08	3.46	3.89	4.06
Chloride (98–108 mmol/l)	117	127	131	130	106	111	104	-	114	112	111	105
Arterial Blood pH (7.35 - 7.45)	7.21	7.14	7.24	7.23	7.31	7.3	-	-	7.26	7.23	7.3	7.39
Arterial Blood PCO2 (35 - 45 mmHg)	26	27	29	33	23	19	-	-	21.6	24.1	29	30.6
Arterial Blood HCO3 (22 to 28 mEq/L))	10.6	9.1	12.5	13.8	12	9.7	-	-	8.6	9.9	13	18.3
T3 (0.9 to 2.8 nmol/L)	0.651	1.9	-	2.01	-	-	-	-	-	-	-	1.91
T4 (12 to 30 pmol/L)	5.15	-	-	-	-	-	-	-	-	-	-	15.73
Thyroid stimulating hormone (0.46–4.7 mlU/l )	46.5	-	-	-	-	16.55	-	15.82	-	-	-	13.7
Urine pH (4.5 to 7.8)	8	-	8	-	-	7.6	-	6.2	-	5.6	-	-

The definitive treatment for her current diagnosis was long-term administration of alkali therapy. Shohl’s solution is a combination of sodium citrate and citric acid (each 5mL contains 5mEq sodium and delivers the equivalent of 5mEq bicarbonate). Shohl's solution was started as 15ml oral solution three times a day, in addition to this an oral potassium tablet of 500mg three times a day was also added for hypokalemia and for hypothyroidism oral thyroxin tablet was continued as 150mcg/day.

On the ninth day of admission, the patient showed marked and swift improvement with the treatment. She was able to walk with support and the next day she could walk without support. The patient was discharged on oral Shohl’s solution 15ml three times a day continued, potassium 500mg tablet three times a day for five days and thyroxine tablet 150mcg/day. On follow-up after 10 days the patient came to the clinic walking by herself without any support. Laboratory investigations were repeated which were all normal (Table 1). On follow-up after three months the patient was much improved and was doing daily life activities without any weakness. KCL was stopped on follow up and Shohl's solution was continued as 15ml three times a day for one year. Our plan is to gradually taper the dose of Shohl's solution, right now the patient is on 15ml two times a day and is on regular follow-up.

## Discussion

Hypokalemic periodic paralysis is a rare condition of muscle weakness that can lead to paralysis and its association with non-autoimmune hypothyroidism along with distal renal tubular acidosis is extremely rare [3]. Distal renal tubular acidosis in hypothyroidism of non-autoimmune etiology was first reported in 1996 which presented as hyperkalemic periodic paralysis [4]. In our case we are reporting hypokalemic periodic paralysis with hypothyroidism of non-autoimmune etiology. In this condition there can be one or more mutations in the calcium, sodium or potassium ion channels. Most common mutations are found in the calcium channel produced by chromosome 1 [5]. This serious, episodic and neurological condition may result in cardiorespiratory failure and death. It may last from hours to several days and paralysis may be localized or generalized. Hypo KPP is often hard to diagnose and the diagnosis can only be made on history, clinical examination and confirmed by serum electrolytes levels and trans-tubular potassium concentration gradient during an acute attack of renal tubular acidosis. DRTA has been reported in patients with thyroid dysfunction before, but the pathophysiology remains unclear, although the DRTA is well documented with different autoimmune diseases including thyroid disease [3]. Very few case reports of hypokalemic periodic paralysis have been published, interestingly triggered by high carbohydrates diet, strenuous exercise, exposure to extreme cold, and psychological excitement. Association with gastroenteritis, diuretic abuse, barter syndrome, adenoma of colon and hyperthyroidism has also been reported in literature [6]. 

Our patient was 30 years old and had clinical and laboratory findings of hypokalemic paralysis, alkaline urine and raised thyroid stimulating hormone (Table 1). There was no history of high carbohydrate diet intake, diuretic abuse, vomiting and diarrhea in this case. The serum potassium level was extremely low in our patient (Table 1). The signs and symptoms resolved after 10 days of hospitalization with thyroxine and potassium replacement. A specific trigger responsible for the acute attack of paralysis could not be identified in our patient, which implies that there have to be other factors as well besides the ones described previously which require further research in this regard. We have come to the conclusion that thyroid hormone deficiency is the cause of tubular dysfunction, because when the patient was put on thyroxine on the first day of admission, she improved biochemically as well as clinically. Effects of hypothyroidism and thyroid hormones in general in impaired renal function have also been studied previously and it is suggested that thyroid function should be routinely checked in cases of renal function impairment [7]. Rare diseases involving multiple systems with an array of signs and symptoms are usually hard to diagnose and treat; our case of hypo KPP with DRTA caused by non-autoimmune hypothyroidism is one of those diseases, and reporting it will benefit physicians whenever they encounter such a rare case in their practice.

## Conclusions

Hypokalemic periodic paralysis with distal renal tubular acidosis caused by non-autoimmune hypothyroidism is extremely rare; its diagnosis is only possible by correlating symptomatology with biochemical markers. Potassium replacement and long-term alkali therapy should be considered for treatment.
